# The Central Autonomic Network and Regulation of Bladder Function

**DOI:** 10.3389/fnins.2019.00535

**Published:** 2019-06-13

**Authors:** Holly Ann Roy, Alexander L. Green

**Affiliations:** ^1^Department of Neurosurgery, Plymouth Hospitals NHS Trust, Plymouth, United Kingdom; ^2^Nuffield Department of Surgical Sciences, Medical Sciences Division, University of Oxford, Oxford, United Kingdom

**Keywords:** bladder, autonomic, periaquecductal gray, multiple system atrophy, Parkinson’s disease

## Abstract

The autonomic nervous system (ANS) is involved in the regulation of physiologic and homeostatic parameters relating particularly to the visceral organs and the co-ordination of physiological responses to threat. Blood pressure and heart rate, respiration, pupillomotor reactivity, sexual function, gastrointestinal secretions and motility, and urine storage and micturition are all under a degree of ANS control. Furthermore, there is close integration between the ANS and other neural functions such as emotion and cognition, and thus brain regions that are known to be important for autonomic control are also implicated in emotional functions. In this review we explore the role of the central ANS in the control of the bladder, and the implications of this for bladder dysfunction in diseases of the ANS.

## Introduction

Normal bladder function in the mature human relies on integration between the autonomic and somatic nervous systems. In disease, and during development, the balance may be altered so that the autonomic nervous system (ANS) plays an increased or altered role in bladder control. However, most studies highlight the role of the ANS in bladder control at the level of peripheral nerve and spinal networks, neglecting the role of supraspinal autonomic nuclei. In this review we describe the anatomic distribution of the central autonomic network (CAN), and then discuss what is known about the supraspinal control of the bladder, and consider the overlap between the CAN and central bladder network. Finally, we consider the implications of central autonomic bladder centers for our understanding of diseases affecting the bladder.

## Supraspinal Neural Organization of Autonomic Control

Collectively, the network of brain areas involved in maintaining and regulating the ANS is known as the ‘central autonomic network’ (CAN). Important progress in our understanding of the anatomy and function of the CAN in the human has been made in recent years, including its role in other neural functions such as emotion and cognition (Critchley and Garfinkel, 2015). Much of this progress is due to developments in neuroimaging techniques (Hyam et al., 2012; Macey et al., 2016). Furthermore, modulation of activity within the CAN at specifically selected anatomic targets, using deep brain stimulation (DBS), offers a possible means of altering or modifying autonomic functions in human patients, and carefully observed studies of autonomic side effects arising from DBS for other indications, have made it possible to experimentally test the effects of DBS on the ANS (Lovick, 2014).

Regulation of autonomic function occurs via the interaction of various brain structures, which contribute to the CAN. Functions of the CAN include regulating the balance of sympathetic and parasympathetic activity at any one time. This regulation can occur as part of feedback loops that are specific to selected organ systems, or secondary to central command mechanisms which are generated by higher cortical areas, for example, as occurs in preparation for exercise (Basnayake et al., 2012). Brainstem sites that are important for autonomic outflow include the locus coeruleus, the parabrachial nucleus of the pons (Loewy, 1991; Napadow et al., 2008), and various nuclei within the medulla including sympathetic outflow pathways in the ventral medulla, the caudal and rostroventral medulla (Loewy, 1982; Gianaros et al., 2012) and parasympathetic outflow nuclei including the nucleus ambiguus and the dorsal motor nucleus (Gianaros et al., 2012). Important subcortical autonomic input nuclei include the nucleus of the solitary tract (NTS) in the medulla, which receives afferent input from the sympathetic and parasympathetic systems, and from baroreceptors and chemoreceptors. The interaction between midbrain, diencephalic and forebrain structures and the brainstem autonomic nuclei are extensive. The role of these regions has been extensively studied in animal recording and lesion studies as well as human cases of stroke and other lesions. Now, autonomic challenges can be applied during functional magnetic resonance imaging (fMRI) to study neural changes associated with autonomic activation in the human. Autonomic challenges include the Valsalva maneuver and handgrip challenges (Saito et al., 1986), forehead, foot or hand cold pressor tests (Macey et al., 2014) and end-expiratory breath hold challenges, which produce sympathetic excitation (Zubin Maslov et al., 2014). These tasks typically induce global changes in blood oxygenation and blood flow in addition to local changes related to increased or decreased neural activation, thus, the global changes are typically excluded from analyses as confounding factors (Macey et al., 2016). fMRI has been particularly useful for investigating the role of cortical structures in autonomic control.

One important cortical region in higher response to autonomic challenges is the insula (Napadow et al., 2008; Al-Otaibi et al., 2010; Critchley et al., 2015; Macey et al., 2016). The insular cortex is buried within the lateral sulcus, and is overlain by and intimately related to, the parietal opercula, whose gyri and sulci interdigitate with those of the insula (Ture et al., 1999). Its activity may affect autonomic outflow via connections to thalamic, limbic and brainstem regions, including the hypothalamus (Allen et al., 1991). Certain fMRI paradigms, using a foot cold pressor challenge, handgrip task (both with predominantly sympathetic components) and Valsalva maneuvre (activating both sympathetic and parasympathetic pathways) have demonstrated a topographical organization of autonomic response within the insula, with an anteroposterior and lateralized differentiation of responses over the 5 gyri of the insula and the two brain hemispheres (Macey et al., 2012) and a unique pattern of activation associated with each task.

The cingulate cortex has also been established as a key region for autonomic response with anterior, mid and posterior cingulate cortex showing differential involvement. For example, posterior cingulate cortex activity has been associated with a withdrawal or reduction in parasympathetic activity (O’Connor et al., 2007; Wong et al., 2007), while anterior cingulate cortex activity has been correlated with sympathetic nervous system activity using pupillary dilatation and skin conductance to infer sympathetic activation (Nagai et al., 2004; Critchley et al., 2005). Stressor-evoked suppression of baroreflex sensitivity was found to co-vary with signal in the perigenual anterior cingulate cortex, dorsal anterior cingulate cortex and posterior cingulate cortex (Gianaros et al., 2012), as well as producing increased functional connectivity between the perigenual ACC and the amygdala, periaqueductal gray area (PAG), and pons.

The hippocampus and amygdala both respond to changes related to activation of the sympathetic nervous system, such as blood pressure challenges (Harper et al., 2000) and tasks evoking effortful stress (Critchley et al., 2000). Their activity has also been shown to correlate with changes in a heart rate variability derived high frequency regressor, which is thought to provide an indication of parasympathetic output to the heart (Napadow et al., 2008). Both amygdala and hippocampus are activated in tasks involving the Valsalva maneuver (Harper et al., 1998; Henderson et al., 2002); the amygdala during hypoxic breathing challenge (Critchley et al., 2015), and the hippocampus in tasks involving inspiratory resistance (Faull et al., 2016). Interestingly, feedback from the ANS modulates activation of these regions in response to an emotional fear task: both amygdala and hippocampus demonstrated a significant main effect of cardiac cycle (systole/diastole) in their response to fearful stimuli (Garfinkel et al., 2014).

Other cortical regions that have been implicated in autonomic function include the ventromedial prefrontal cortex (Verberne et al., 1997), and sensorimotor cortices (pre- and post- central gyrus). Autonomic activation of sensorimotor cortex is difficult to dissociate from task based motor and sensory activation, however, Nowak et al. (1999) demonstrated increased activation of post-central gyrus with a hand grip exercise that persisted after the application of local anesthesia, supposedly ruling out a direct sensory input to the region, and activation of the post-central gyrus has also been described during heart rate changes in a task designed to elicit baroreceptor activation alone independent of motor or cognitive effort by inducing baroreflex responses using lower body negative pressure (Kimmerly et al., 2005). Ventromedial prefrontal activity has been show to vary with skin conductance level (Nagai et al., 2004) and stimulation of muscle sensory afferents (Goswami et al., 2011) and it has been suggested that its activity is related to hippocampal activation.

Finally, the cerebellum appears to have an important role in autonomic control. The cerebellum responds to blood pressure changes that occur in a variety of tasks including Valsalva (Harper et al., 1998; Henderson et al., 2002), ventilatory challenges of 5 and 10% CO_2_ (Harper et al., 1998), changes in minute ventilation (Critchley et al., 2015), heart rate changes (Gray et al., 2007) forehead and hand cold pressor tasks (Harper et al., 1998), a moderate intensity handgrip task (Wong et al., 2007), and emotional arousal (O’Connor et al., 2007). Activation of the cerebellar vermis specifically has been reported during effortful tasks designed to elicit a stress response (Critchley et al., 2000) as well as emotional arousal (O’Connor et al., 2007), moderate intensity handgrip (Wong et al., 2007) and blood pressure increases (Critchley et al., 2015).

## Supraspinal Organization of Bladder Conrol and Overlap With the Central Autonomic Network

**FIGURE 1:**
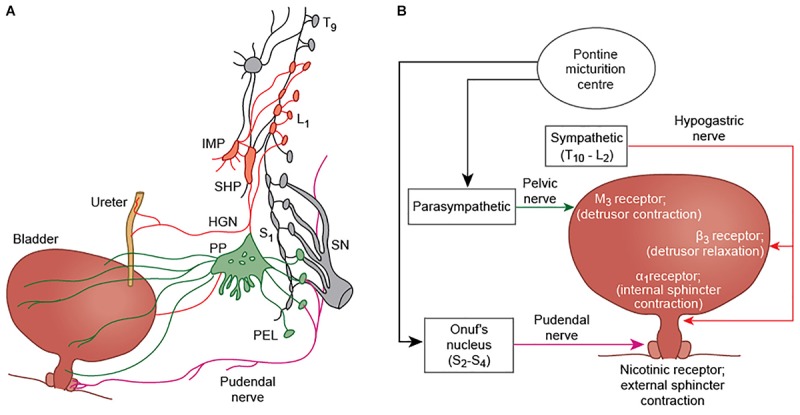
**(A)** Efferent innervation of the bladder, ureter, and external urethral sphincter (modified from Fowler et al., 2008). Parasympathetic fibers (green) arise from S2-4 nerve roots. They travel in the pelvic nerve and pass via the pelvic plexus to innervate the bladder detrusor muscle of the bladder. Sympathetic fibers (red) arising from T11-L2 segments, pass via the inferior mesenteric plexus (IMP) and travel in the hypogastric nerve (HGN) to innervate the ureter and the detrusor smooth muscle. Somatic innervation of the external urethral sphincter is via the pudendal nerve, and arises from Onuf’s nucleus at S2-S4. **(B)** A simplified schematic showing the major nerves and neurotransmitters involved in bladder control. The pudendal nerve causes contraction of the external urethral sphincter via nicotinic receptors (+N); parasympathetic fibers produce contraction of the detrusor muscle by acting at M3 receptors; and sympathetic innervation inhibits detrusor contraction through β3 receptors, and contraction of the internal urethral sphincter by α1 receptor activation.

The central nervous system plays a critical role in the regulation of the urinary bladder. Bladder control involves various autonomic, cognitive and sensorimotor operations, and establishing an understanding about the functional role of different brain regions in bladder circuitry, and how these regions interact with each other and with the peripheral nervous system, has been a key goal of neuro-urological research. The bladder is somewhat unique, in that while it is regulated by autonomic networks at the level of the spinal cord (and in the human infant is entirely under autonomic control) there is conscious awareness of bladder filling and voluntary control over the process of micturition. Moreover, in many non-human animals, there are major social functions of urination besides expulsion of waste products from the body (Hou et al., 2016). By default, the lower urinary tract remains in “storage” mode during bladder filling- in this state, the external urethral sphincter must remain contracted and the detrusor muscle relaxed to facilitate urine collection. It is thought that a set of brain areas are active in the storage state to maintain these properties of the detrusor and sphincter, but also to monitor the progression of bladder filling, whether in a conscious or subconscious fashion. When the bladder becomes full, there is a “switch” (requiring another set of neural events) to “voiding” mode. The “voiding” mode is the state in which the bladder sphincters relax and the detrusor muscle contracts, permitting micturition, and calls into play a third distinct pattern of brain activity. The mechanistic control of the bladder detrusor muscle and sphincters occurs through the activity of autonomic and somatic networks in the spinal cord, which communicate reciprocally with supraspinal centers, although a direct connection between the pontine micturition center (PMC) and sympathetic preganglionic neurons has there has not currently been demonstrated. There are two separate circuits for bladder control; bladder storage pathways which exist entirely within the spinal cord itself, and the supraspinal pathway which is thought to maintain continence when the bladder is significantly full, and which in non-human animal species may also be important for the social functions of micturition. Parasympathetic nerves (otherwise known as pelvic splanchnic nerves) are derived from the S2-4 nerve roots, which pass via the pelvic plexus and trigger bladder contraction during voiding. Sympathetic neurons have the effect of maintaining bladder relaxation, and travel along the iliohypogastric nerve (derived from T10-L2 roots, formed in the lumbar plexus). Somatic cholinergic nerves arising in Onuf’s nucleus (located at S2-4 spinal level) travel in the pudendal nerve (formed from ventral rami of S2-4 and the coccygeal nerve in the sacral plexus) to innervate the external urethral sphincter; Onuf’s nucleus is likely to be innervated by the pontine L region or pontine continence center, which is separate to the PMC and important for maintaining continence during times of urgency (Griffiths et al., 1990). Sensory information about bladder fullness is carried along the pelvic (S2-4) and iliohypogastric nerves (T12-L1), while sensory information from the bladder neck and urethra is transmitted by the pudendal nerve and iliohypogastric nerve (Fowler et al., 2008) (see Figure 1 for afferent and efferent nerves involved in bladder control). In the spinal cord, animal studies suggest that bladder afferents travel in Lissauer’s tract before projecting in lamina I, V–VII, and X of the dorsal horn (de Groat and Yoshimura, 2010). Parasympathetic preganglionic neurons, responsible for detrusor contraction, receive projections from the lateral and dorsolateral funiculus, the dorsal gray commissure, and lamina I of the dorsal horn (likely transmitting primary afferent bladder information) (de Groat and Wickens, 2013).

To understand the supraspinal control of the lower urinary tract, various experimental approaches have been used. Animal studies, using tract tracing, electrical stimulation, lesioning, and neural recording techniques were instrumental in identifying key subcortical regions important for bladder control, including the PMC which represents the major outflow tract to initiate micturition (Loewy et al., 1979; Holstege et al., 1986; Blok and Holstege, 1997), the pontine continence center (Holstege et al., 1986; Griffiths et al., 1990), the PAG, which receives afferent information about bladder filling and may be significant in switching from storage to voiding mode (Noto et al., 1991; Blok et al., 1995; Liu et al., 2004), and the globus pallidus (Lewin and Porter, 1965; Porter et al., 1971), amongst many others. Of particular relevance to the ANS, the role of the locus coeruleus (LC) in bladder control has been demonstrated (Valentino et al., 1996); the LC is activated along with the PMC, by PAG stimulation (Meriaux et al., 2018) and displays neuronal activity that temporally correlates with the initiation of micturition (Manohar et al., 2017). More recently, newer techniques such as optogenetics and fiber photometry have built on earlier studies to deepen insights into the supraspinal pathways involved in bladder control. For example, Hou et al. (2016) used optogenetic techniques to identify a population of corticotrophin-releasing hormone (Crh) positive neurons within the mouse PMC whose activity correlated with bladder contractions. These neurons were found to send glutamatergic projections to the spinal cord, consistent with an excitatory role, and, using rabies-based retrograde trans-synaptic labeling, the group identified widespread connections from higher brain centers potentially capable of modulating activity of the Crh-positive neuronal population. These connecting regions included areas regarded as important for autonomic control, such as the anterior cingulate cortex, hypothalamus, central amygdalar nucleus, and PAG; as well as other regions such as the motor and somatosensory cortex. Similarly, Keller et al. (2018) used optogenetics to identify a subset of neurons within Barrington’s nucleus expressing estrogen receptor 1 which may be involved in relaxing the external urethral sphincter.

Despite a good understanding of bladder control networks in animal models, confirming similar networks in the human have traditionally been challenging. Early insights into bladder control networks in humans were inferred through studies of patients with brain lesions that impaired urinary function (Andrew and Nathan, 1964). Important areas identified by this approach included the frontal lobes in general (Maurice-Williams, 1974), and brainstem regions including the PAG (Yaguchi et al., 2004). More recently, alterations in bladder function have been reported following implantation of DBS electrodes within the thalamus (Kessler et al., 2008), subthalamic nucleus (STN; Seif et al., 2004; Herzog et al., 2006, 2008), internal globus pallidus (GPi) (Mordasini et al., 2014), PAG (Green et al., 2012) and pedunculopontine nucleus (PPN; Aviles-Olmos et al., 2011; Roy et al., 2018b), implicating all of these regions in some aspect of human urinary control. Finally, developments in functional brain imaging have facilitated a controlled, systematic, experimental whole-brain approach to investigate the neural control of the bladder in both healthy human subjects and those with bladder disorders. Functional imaging experiments are able to identify patterns of neural activation that occur in response to specific bladder-related tasks or states, including urinary voiding, bladder filling or the subjective experience of certain bladder-related sensations, such as urinary urgency. Although a range of functional imaging methodologies have been used including positron emission tomography (PET; e.g., Blok and Holstege, 1997; Nour et al., 2000), fMRI (fMRI; e.g., Griffiths et al., 2005; Mehnert et al., 2008) and near infrared spectroscopy (Matsumoto et al., 2009), fMRI, as a relatively safe and non-invasive technique, with good spatiotemporal resolution, is rapidly becoming the dominant modality.

The most common type of paradigm used for brain-bladder fMRI is an “infusion withdrawal” paradigm (see Griffiths et al., 2005), whereby many repetitions of infusion and withdrawal of small volumes of fluid into the bladder are carried out, usually at a relatively empty bladder and a relatively full bladder (in many cases, the bladder is filled to provoke the sensation of urgency). The contrast (infusion-withdrawal) is averaged over multiple trials, due to the inherently low signal-to-noise of fMRI, and is thought to reveal brain activations associated with bladder filling or the experience of urinary urgency (depending on the exact details of the trial). Other approaches using fMRI to study brain-bladder activity include measuring the BOLD signal during or immediately preceding urinary voiding (Krhut et al., 2012; Shy et al., 2014) and, more recently, the use of resting state fMRI (Nardos et al., 2014) in the full and empty bladder state.

**FIGURE 2:**
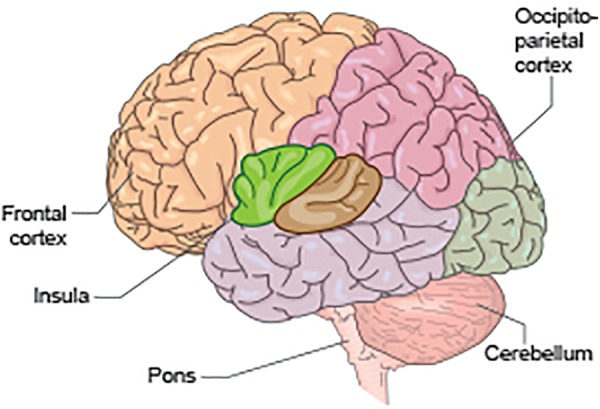
Schematic to illustrate some of the key anatomical regions significant for bladder control, including the pons, cerebellum, insula, occipito-parietal cortex, and frontal cortex.

**FIGURE 3:**
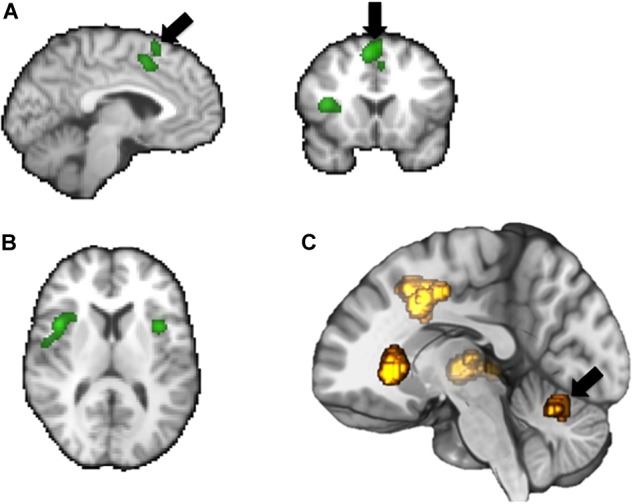
Summary of some key brain areas demonstrated to be involved in bladder related tasks based on fMRI: supplementary motor area (SMA), mid-cingulate gyrus **(A)**, frontal operculum **(B)**, insula **(C)**, cerebellum.

fMRI studies designed to elicit brain activations relating to the “storage” mode have demonstrated activations in regions including frontal cortex, anterior cingulate cortex, insula, parahippocampal gyrus and cerebellum, as well as activations in the thalamus and brainstem (Mehnert et al., 2008; Tadic et al., 2010, 2013; Krhut et al., 2014). Voiding-related fMRI activity occurs predominantly within the bilateral cingulate cortex and bilateral medial frontal cortex (i.e., SMA), occipito-parietal regions, insula, parahippocampal gyrus, and pons (Krhut et al., 2012; Shy et al., 2014) (see Figure 2,3).

A current integrative model for the neural control of the bladder summarizes bladder control in terms of three separate ‘circuits’ of brain areas, which converge on a central hub (Griffiths, 2015). Circuit 1 involves the thalamus, insula, and lateral prefrontal cortex (connecting to the medial prefrontal cortex which regulates voiding) and is modeled as the normal circuitry for handling bladder sensation. Circuit 2 involves the dorsal anterior cingulate cortex (dACC) and supplementary motor area (SMA), important in creating a sensation of urgency and controlling pelvic floor muscles to prevent accidental urinary leakage in patients with urge urinary incontinence. Circuit 3 is thought to involve the parahippocampal gyrus although its precise functional role is not described in detail.

Despite our growing knowledge of brain activity associated with urine storage and voiding, few frameworks have been created to help conceptualize which parts of these bladder functional networks relate to autonomic control. Indeed, most thinking about autonomic control of the bladder relates to the peripheral bladder storage pathways which exist at the level of the spinal cord. However, considering our present understanding of the central bladder network, and incorporating separate research on central autonomic control, brain areas common to both include the insula, cerebellum, and cingulate cortex. The insula is thought to be the primary cortical area for the representation of visceral interoception, receiving afferent information from Aδ and C fibers and creating a subjective awareness of an individual’s homeostatic state (Craig, 2003), including with regard to bladder filling, and from an autonomic perspective, is thought of as an autonomic sensorimotor region (Hyam et al., 2012), playing a key role in autonomic challenges including blood pressure challenges (Macey et al., 2016). The cingulate cortex is activated during both storage and voiding, and in the model above may also be linked to the experience of urinary urgency. This fits with the view that the ACC is the brain’s homeostatic motor area (Craig, 2003), regulating motor and autonomic responses to maintain internal balance (Critchley et al., 2003), including, perhaps, regulating urinary voiding when necessary. Cerebellar activation is frequently reported during bladder filling in PET and fMRI (e.g., Nour et al., 2000; Athwal et al., 2001; Matsuura et al., 2002) as well as in various autonomic challenges such as Valsalva (Harper et al., 2000) and static hand grip testing (Macefield and Henderson, 2015). Interestingly, without deliberate reference to autonomic control, it has been proposed that the cerebellum, anterior insula, and cingulate cortex are functionally part of a common network, which may have an attentional role (Jarrahi et al., 2015). Studies that combine autonomic function tests with bladder filling or urinary voiding paradigms in the fMRI scanner are the next step in defining more completely to what extent these brain regions are playing an “autonomic” role in bladder control.

It may also be that brainstem regions important for bladder control but difficult to resolve with current fMRI techniques due to technical challenges of brainstem imaging, provide the critical link between somatic and autonomic systems regulating the bladder. For example, the raphe nuclei, the noradrenergic cell population in the locus coeruleus and the A11 dopaminergic cell group connect with autonomic and somatic motorneurons and also project to bladder motorneurons and Onuf’s nucleus (Holstege, 2005). Similarly, the paraventricular nucleus of the hypothalamus (also difficult to resolve using neuroimaging) has projections both to autonomic motoneurons and to Onuf’s nucleus (Holstege, 1987), while the PAG has major effects on autonomic control (Lovick, 1992; Green et al., 2010) and fight or flight responses (These regions may be pivotal in understanding the supraspinal control of the bladder and integration of somatic and autonomic control mechanisms.). However, although newer studies are providing insights into the activity of specific sub-populations of cells within brain areas and bladder function in animal models, this level of specificity is yet to be achieved in human studies although newer technologies such as 7T MRI scanning and local field potential recordings from implanted depth electrodes (Roy et al., 2018a) may help to isolate smaller neuronal populations.

## Autonomic Disorders and Effects on Bladder Control

A number of neurodegenerative diseases with a clear autonomic component are associated with lower urinary tract symptoms. Research is needed to clarify the exact mechanism giving rise to bladder dysfunction in these conditions, and to determine whether the symptoms are a direct consequence of damage to autonomic bladder circuits or associated parts of the brain-bladder network.

### Parkinson’s Disease

Parkinson’s disease is a neurodegenerative disease characterized by loss of dopaminergic neurons within the substantia nigra, and aggregation of alpha-synuclein deposits as Lewy bodies in neurons. The result is a prominent motor disorder accompanied by autonomic and cognitive symptoms. Common autonomic symptoms in Parkinson’s disease include orthostatic hypotension, swallowing difficulties, sweating, constipation, diarrhea, and urinary storage symptoms including frequency, urgency, and nocturia (Sakakibara et al., 2001; Chaudhuri et al., 2006; Jain, 2011). Bladder symptoms are experienced by 38–71% of patients with PD (Sakakibara et al., 2018). Peripheral nerve and peripheral organ involvement appears to contribute to autonomic symptom development in PD; most convincingly, cardiac sympathetic denervation has been associated with orthostatic hypotension (Goldstein et al., 2002; Goldstein et al., 2007), while links have also been made with peripheral Lewy body pathology and associated autonomic symptomatology in other organ systems, particularly the enteric nervous system (Lebouvier et al., 2010; Cersosimo and Benarroch, 2012). However, despite the demonstration of peripheral nervous system involvement in the onset of autonomic symptoms, central nervous system abnormalities are also likely to be implicated. Brain structural abnormalities that underlie these common autonomic symptoms in Parkinson’s disease have not been conclusively investigated, although hypothetically, it might be expected that structural alterations within the CAN, such as the ventromedial medulla, hippocampus, insula, cingulate cortex, ventromedial prefrontal cortex, and cerebellum (Macey et al., 2016) could be implicated. Chen et al. (2016) described reduced baroreflex sensitivity and reduced gray matter in the left hippocampus, right amygdala, bilateral insula, bilateral cerebellum, bilateral caudate, right fusiform, and left middle frontal gyrus in patients with Parkinson’s disease (Chen et al., 2016).

Bladder symptoms in Parkinson’s disease are predominantly storage-related, producing a clinical picture of overactive bladder (Sakakibara et al., 2018). Urodynamic features include reduced bladder capacity, detrusor overactivity (Sakakibara et al., 2001), uninhibited sphincter relaxation and reduced detrusor contractility during voiding (Terayama et al., 2012). It is thought that overactive bladder symptoms might relate to the primary loss of dopaminergic neurons in the substantia nigra and the reduction in the inhibitory influence of dopamine acting at D1 receptors and interacting with bladder circuitry (Kitta et al., 2008), however altered function of CAN centers as a result of Lewy body pathology may also be significant. However, there are limited functional or structural MRI studies which investigate bladder symptoms in PD, and although urinary symptoms are often classified as an autonomic feature of PD, the specific neuroanatomical correlates for this classification have not yet been confirmed.

### Multiple System Atrophy

Like PD, multiple system atrophy (MSA) is characterized by alpha-synuclein aggregates. However, in MSA, these develop within the oligodendrocytes rather than neurons and are known as glial cytoplasmic inclusions. The alpha-synuclein pathology tends to be more widespread than in PD and includes involvement of the spinal cord. Bladder dysfunction in MSA occurs at an earlier stage in disease than in PD, and tends to be more severe. Over 90% of patients with MSA have some form of lower urinary tract symptoms (Yamamoto et al., 2009, 2011). Moreover, prominent voiding symptoms, characterized by urinary retention, are observed in addition to symptoms of overactive bladder (Sakakibara et al., 2018). Detrusor underactivity, which significantly contributes to voiding difficulties in MSA, may result from degeneration in the PMC, whereas overactive bladder symptoms are more likely to result from degenerative changes in the locus coeruleus, pontomedullary raphe (Ito et al., 2006), and cerebellar vermis (Sakakibara et al., 2004), all of which have been identified as brain regions important for autonomic control. Alongside changes within the brain, spinal, and peripheral neurodegeneration also occurs which may contribute to symptoms. In particular, Onuf’s nucleus in the anterior horn of the sacral spinal cord, which contains the somatic motor supply to the external urethral sphincter, undergoes neuronal cell loss in MSA, and although there is not always a clear correlation between symptoms and neurogenic changes on EMG, weakness of the sphincter can result in incontinence in female MSA patients. There is also thought to be loss of sympathetic innervation of the bladder neck due to degeneration at the intermediolateral nucleus, resulting in a high incidence of open bladder neck.

### Other Central Autonomic Disorders

Various other autonomic disorders such as postural tachycardia syndrome (POTS) (Kaufman et al., 2017) and pure autonomic failure (PAF) (Singer et al., 2017) also have associated bladder dysfunction. This reflects the intimate relationship between the lower urinary tract system and the ANS.

### Emotional and Affective Disorders

Given the overlap between “limbic” neurocircuitry and both autonomic and bladder control regions, we were interested to identify whether there is evidence for bladder dysfunction in emotional or affective disorders. Research from the animal literature demonstrates a link between social stress in rats (Wood et al., 2009) and mice (Chang et al., 2009; Mingin et al., 2014) and changes in bladder physiology. For example, Wood et al. (2009) found that in rats subjected to social defeat, there was a significant increase in bladder to body weight ratio, which correlated inversely with the latency to adopt the defeat posture during social stress. Rats in the social defeat group also demonstrated changes in the pattern of micturition including reduced frequency of voiding and altered voiding pattern (voiding in the corner of the cage) compared with control animals, which maintained the same number of voiding spots as at the start of the experiment and also continued to void throughout the cage. Increased expression of corticotropin releasing factor in neurons of Barrington’s nucleus in the social stress group was proposed as a possible mechanism mediating the changes in bladder function.

From human clinical studies, however, there is surprisingly little research to investigate the relationship between bladder control and stress, trauma or emotional/affective disorders. This is likely to be in part due to the challenges associated with developing an appropriate and ethical experimental paradigm in humans. Nevertheless, it appears that trauma may have a profound impact on bladder control, particularly childhood trauma. For example, Durkin et al. (1993) carried out a population-based neurodevelopmental study of children in a rural community in Bangladesh. 6 months later, there was a devastating flood, the most severe ever recorded at that point in Bangladesh. Following this, a subset of the surviving children were re-evaluted. The group found that lack of sphincter control (bowel/bladder) had increased from 16.8% at baseline to 40.4% following the flood, a change which was statistically significant (Durkin et al., 1993). There was also a significant increase in aggressive behavior. Similarly, in a study comparing adult patients with interstitial cystitis/bladder pain syndrome with healthy controls presenting with acute cystitis, Chiu et al. (2017) identified higher rates of physical abuse in childhood or adulthood, and higher rates of childhood trauma by close others, in the IC/BPS group (Chiu et al., 2017). In a prospective study of female veterans who had returned from active service, Bradley et al. (2017) identified baseline anxiety, post-traumatic stress disorder and lifetime history of sexual assault as predisposing factors associated with the development of new overactive bladder syndrome at 1 year follow up. Clearly, the link between emotional/affective disorders, particularly PTSD and bladder dysfunction, needs further investigation with neuroimaging involvement to understand the link between these conditions more deeply, however, it is likely that common substrates to autonomic and bladder control such as the anterior cingulate cortex (Rinne-Albers et al., 2017), insula (Perez et al., 2017), and PAG (Johansen et al., 2010; Nicholson et al., 2017) may be involved.

## Conclusion

Research into the central organization of autonomic control and bladder control have until now progressed relatively independently. However, there is clear overlap between supraspinal networks regulating autonomic and bladder function, with implications for the development of lower urinary tract symptoms in disease. Further research to explore the specific autonomic role of known areas in the bladder control network is needed, as is detailed investigation of the structural and functional brain changes associated with urinary symptoms in autonomic and emotional/affective disorders.

## Author Contributions

All authors listed have made a substantial, direct and intellectual contribution to the work, and approved it for publication.

## Conflict of Interest Statement

The authors declare that the research was conducted in the absence of any commercial or financial relationships that could be construed as a potential conflict of interest.
